# Case Report: Dual pathogenic mechanism of a *PRKG2* missense variant underlies an attenuated phenotype of acromesomelic dysplasia

**DOI:** 10.3389/fgene.2025.1708985

**Published:** 2026-01-08

**Authors:** Daria Akimova, Tatiana Markova, Maria Orlova, Vladimir Kenis, Mikhail Skoblov

**Affiliations:** 1 Research Centre for Medical Genetics, Moscow, Russia; 2 H. Turner National Medical Research Center for Children’s Orthopedics and Trauma Surgery, Saint Petersburg, Russia; 3 North-Western State Medical University named after I. I. Mechnikov, Saint Petersburg, Russia

**Keywords:** acromesomelic dysplasia, dwarfism, *PRKG2* gene, RNA analysis, splicing variant

## Abstract

Acromesomelic dysplasia comprises a group of rare skeletal disorders characterized by dwarfism with anomalies predominantly affecting the middle and distal segments of the limbs. Based on genetic variants, five types are recognized, with significant phenotypic variability even within a single type. Here, we describe a girl presenting with borderline short stature and mild disproportion due to acro- and mesomelic shortening of the limbs. Radiographic examination revealed shortening of the radius and ulna, mild brachydactyly, absence of iliac flaring and metaphyseal alterations of the long bones, and biconcave appearance of the femoral necks and II-IV metacarpals, and an elongated styloid process of the ulna. Using WGS, we identified two novel variants in the *PRKG2* gene: a frameshift variant (NM_006259.3:c.1074del (p.Ala359LeufsTer24)) and a missense variant (NM_006259.3:c.1630G>T (p.Asp544Tyr)). Functional analysis unveiled a unique dual pathogenic mechanism: the missense variant creates a cryptic splice site, resulting in two aberrant protein products - an in-frame deletion and a missense substitution. We hypothesize that these alterations cause a partial, rather than a complete, loss of protein function, which may account for the patient’s attenuated clinical phenotype.

## 1 Introduction

Acromesomelic dysplasia (AMD) is a group of autosomal recessive skeletal disorders characterized by dwarfism associated with anomalies of the middle and distal segments of the extremities (Khan et al., 2016). Radiographic features typically include shortening and broadening of long bones, metaphyseal changes, and vertebral anomalies. The disease is notable for both significant clinical and genetic heterogeneity. The five types of AMD are classified according to the underlying gene in which pathogenic variants occur: the severe Grebe dysplasia (Costa et al., 1998) and Hunter-Thompson dysplasia (Langer et al., 1989; Thomas et al., 1996), and the milder du Pan dysplasia (Szczaluba et al., 2005), Maroteaux type (Simsek-Kiper et al., 2021), and the PRKG2-linked type (Díaz-González et al., 2022).

More recently, biallelic variants in *PRKG2*, encoding cGMP-dependent protein kinase II (PKG II), have been identified as a novel cause of acromesomelic dysplasia (Kumar et al., 2021; Díaz-González et al., 2022; Pagnamenta et al., 2022; Mollaoğlu et al., 2023; Akgun-Dogan et al., 2024). This *PRKG2*-associated form shares many clinical and radiological features with Acromesomelic dysplasia, type Maroteaux. *PRKG2* plays a pivotal role in chondrocyte differentiation, longitudinal bone growth, and endochondral ossification through its regulation of MAPK signaling downstream of C-type natriuretic peptide (CNP)–NPR2 activation (Koltes et al., 2015). Loss-of-function variants in *PRKG2* impair this signaling cascade, leading to defective growth plate organization and reduced skeletal growth (Miyazawa et al., 2002; Tsuchida et al., 2008).

To date, seven pathogenic point variants in the *PRKG2* gene have been described in patients with acromesomelic dysplasia in the ClinVar and HGMD databases: one missense variant NM_006259.3:c.1409T>G (p.Val470Gly) (ClinVar accession number: VCV002501812.2) (Akgun-Dogan et al., 2024), two nonsense variants NM_006259.3:c.764G>A (p.Trp255Ter) and NM_006259.3:c.1705C>T (p.Arg569Ter) (ClinVar accession number: VCV001326257) (Díaz-González et al., 2022; Pagnamenta et al., 2022), two frameshift variants NM_006259.3:c.491dup (p.Asn164Lysfs*2) (ClinVar accession number: VCV001326258) (Díaz-González et al., 2022) and NM_006259.3:c.2282dup (p.Asp761Glufs*34) (ClinVar accession number: VCV001326259) (Pagnamenta et al., 2022), and two variants located in canonical splice sites, NM_006259.3:c.1635-1G>C (Mollaoğlu et al., 2023) and NM_006259.3:c.1154+1G>A (ClinVar accession number: VCV002502276) (Akgun-Dogan et al., 2024). Thus, the majority of the identified variants are loss-of-function (LoF) variants, while the missense variant has been identified and described only once (Akgun-Dogan et al., 2024).

We describe the clinical and molecular features of a female proband with a mild form of acromesomelic dysplasia, in whom we identified two novel compound-heterozygous variants in the *PRKG2* gene: a LoF variant NM_006259.3:c.1074del (p.Ala359LeufsTer24) (ClinVar accession number: VCV004537385.1) and a missense variant NM_006259.3:c.1630G>T (p.Asp544Tyr) (ClinVar accession number: VCV004082151.1). Our functional studies using targeted next-generation sequencing (NGS) of RT-PCR products demonstrated the impact of these variants on the structure and expression of *PRKG2* mRNA and allowed us to establish the molecular cause of the proband’s milder clinical presentation.

## 2 Materials and methods

### 2.1 Patient

Patient recruitment for this study was conducted in accordance with the Declaration of Helsinki, and approval was obtained from the Institutional Review Board of the Research Centre for Medical Genetics. The patient and her relatives provided informed consent for clinical examination, molecular studies, and publication of the anonymized data.

### 2.2 WGS

WGS data processing was carried out using the “NGSDataGenome” program (Beskorovainy NS. Program “NGSData” // Certificate of State Registration of Computer Programs No. 2021662119.2021). For the *PRKG2* gene, RefSeq accession number NM_006259.3 was used.

### 2.3 Sanger sequencing

Validation of the variants identified through WGS was performed using Sanger sequencing. DNA samples from the proband’s and his parents’ peripheral blood were used as templates. For the validation and segregation of the NM_006259.3:c.1074del (p.Ala359LeufsTer24) variant, the following primers were used 5′-TGT​TGC​CAT​TAA​CTT​GGT​AGC​TCA-3′ and 5′-AGG​GTT​TGG​TTG​AAT​GAA​TGT​GTG​T-3’. For the validation and segregation of the NM_006259.3:c.1630G>T (p.Asp544Tyr) variant, the primers 5′-TCC​TAA​TGA​TTT​CCT​CTA​TGC​C-3′ and 5′-TGG​AGA​TGA​GTA​TCA​TTG​TTC​C-3′ were used.

### 2.4 RNA analysis

An analysis of the mRNA structure of the *PRKG2* locus, harboring the NM_006259.3:c.1630G>T (p.Asp544Tyr) and NM_006259.3:c.1074del (p.Ala359LeufsTer24) variants, was performed using RNA isolated from dermal fibroblasts acquired from the proband and the proband’s parents. Primary fibroblast cultures were obtained from patient skin biopsies at the Moscow Branch of the All-Russian Collection of Biological Samples of Hereditary Diseases Biobank. Total RNA extraction was carried out utilizing the Extract RNA reagent (Evrogen, Russia), followed by cDNA synthesis with the Reverse Transcription System (Dialat, Russia) and oligo-dT primers in accordance with manufacturer’s protocol. cDNA quality was assessed through quantitative PCR amplification of the *B2M* reference gene. The target locus was amplified employing primers 5′-AAG​GAG​ATT​ACA​TCA​TTA​GAG​AGG​G-3′ and 5′-ATT​TAA​GGT​AAC​CCT​CAG​CAT​CT-3′, with subsequent deep sequencing analysis.

### 2.5 Targeted next-generation sequencing of RT-PCR product

For targeted next-generation sequencing, libraries were prepared using the SG GM Kit (Raissol) and sequenced on the FASTASeq platform in paired-end mode (2 × 150 bp), achieving greater than 8,000× coverage depth across the target locus in all samples. Raw sequencing data underwent processing through a custom analytical pipeline incorporating open-source bioinformatics tools, including quality control assessment with FastQC v0.12.1, read alignment to the hg38 reference genome using STAR v2.7.11b, and splice junction visualization via Sashimi plot implementation in IGV.

### 2.6 3D protein analysis

3D structural analysis of the cGMP-dependent protein kinase 2 was performed using the predicted structure AF-Q13237-F1 generated by AlphaFold2. The resulting model was visualized with the molecular graphics program PyMol (version 2.5.8).

## 3 Results

### 3.1 Clinical description

A 9-year-old girl was referred to a geneticist due to progressive growth retardation. The child of healthy, consanguineous parents (second cousins) has two unaffected older brother and sister. Examination revealed the following heights: a 26-year-old sister measured 158 cm (−0.67 SD), while her two brothers, aged 22 and 15 years, measured 168 cm (−1.1 SD) and 163 cm (−1.1 SD), respectively. The mother’s height is 152 cm, and the father’s is 178 cm. Late-term ultrasonography revealed shortening of the long tubular bones. The delivery was performed via cesarean section at 38–39 weeks of gestation. Birth weight was 3,380 g (0.21 SD), length was 48 cm (−0.72 SD) and the Apgar score was 8/9. Psychomotor development was age-appropriate, and independent walking achieved at 11 months. The parents first noticed growth retardation at the age of 6 months and sought medical advice from a pediatrician at 1 year of age, presenting with the complaint of growth failure. Anthropometric records indicated a height of 70 cm (−2.81 SD) at 1 year and 110 cm (−3.0 SD) at 8 years. Consequently, the proband was referred for an endocrinologist, which identified disproportionate stature due to limb shortening, reduced growth velocity, and a bone age delay of 1.5 years, as evidenced by hand radiography. Chromosome analysis on peripheral blood showed a normal 46, XX female karyotype. Levels of TSH, free T4, IGF-1, parathyroid hormone, ionized calcium, and 25-hydroxyvitamin D were within the normal range.

**FIGURE 1:**
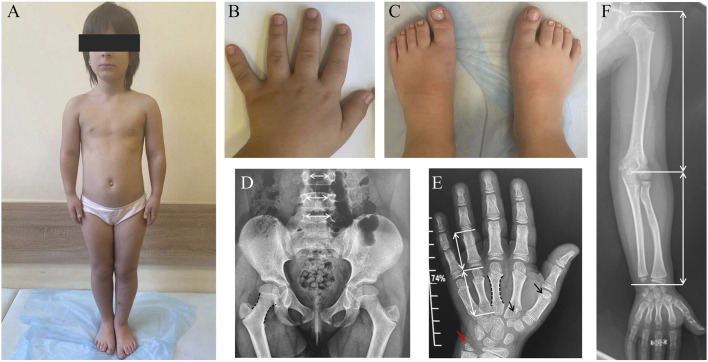
Clinical features of the patient with *PRKG2*-associated acromesomelic dysplasia. **(A)** General appearance showing disproportionate short stature. **(B)** Hands with mild brachydactyly and broad, shortened fingers. **(C)** Feet demonstrating brachydactyly, large and broad great toes and a sandal gap. **(D)** Antero-posterior radiograph of the pelvis and lumbar spine: in general, the proportions of the spine, pelvic and femoral bones are not disturbed; femoral necks appear slightly elongated, slender and bi-concave (black dashed lines); the interpedicular distances from L3 to L5 progressively increase (normal pattern). **(E)** Postero-anterior radiograph of the hand: shortening of the metacarpals relative to the phalanges (white arrows); slender, bi-concave contour of the II-IV metacarpals (black dashed lines); incomplete pseudo-epiphyses of the first and second metacarpals (black arrows); elongated styloid process of the ulna (red arrow). **(F)** Mild mesomelic disproportion in the lengths of the humerus and the forearm (white arrows).

Examination at the age of 9 years revealed moderate short stature with a height of 118 cm (−2.81 SD), weight of 24 kg (−1.71 SD), and head circumference of 51 cm (−0.88 SD). Disproportionate short stature is one of the hallmarks of different types of skeletal dysplasias (Markova et al., 2023). The clinical findings included a prominent forehead, a broad chest, mild lumbar hyperlordosis, shortened forearms, mild brachydactyly of the hands and feet, relatively large and broad great toes and a sandal gap (Figures 1A–C).

Radiographic evaluation at age nine revealed multiple skeletal abnormalities (Figures 1D–F).

Based on the clinical and phenotypic findings, hypochondroplasia was initially suspected. However, no pathogenic variants in the *FGFR3* gene were identified. Subsequently, a targeted next-generation sequencing panel of 166 genes associated with skeletal dysplasias was performed, which also failed to identify the etiology.

### 3.2 Molecular genetic results

**FIGURE 2:**
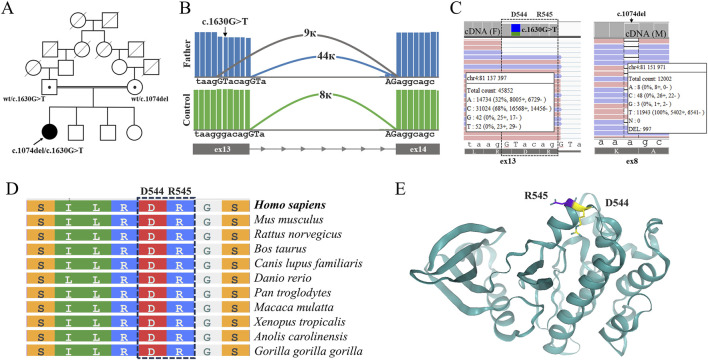
Molecular findings in the proband’s family. **(A)** Pedigree of the family showing segregation of the two variants: the proband is compound heterozygous for NM_006259.3:c.1074del (p.Ala359LeufsTer24) and NM_006259.3c.1630G>T (p.Asp544Tyr), inherited from the mother and father, respectively. **(B)** Sashimi plots demonstrating aberrant splicing associated with the NM_006259.3:c.1630G>T variant in the proband’s father. The variant creates a cryptic splice donor site, leading to a 6-nucleotide shortening of the exon 13 transcript. The values 9 k, 44 k, and 8 k represent the junction counts, which quantify the splicing events occurring between their respective splice sites. The control is mRNA isolated from skin fibroblasts of a healthy volunteer. **(C)** IGV screenshots of targeted deep cDNA sequencing from the father (left) and the mother (right). The NM_006259.3c.1630G>T substitution was confirmed in the father’s sample and showed concurrent expression of two aberrant transcripts: one carrying a truncated exon 13 and another retaining the NM_006259.3:c.1630G>T (p.Asp544Tyr) missense change. The deleted amino acids are highlighted with a dashed line, p.Asp544_Arg545del. The maternal NM_006259.3:c.1074del (p.Ala359LeufsTer24) variant shows allelic imbalance with marked decrease in expression of the mutant allele. The data indicating the number of reads for each nucleotide at the given position are shown based on the minus strand. **(D)** Structural modeling of *PRKG2* kinase domain highlighting the residues Asp544 and Arg545 affected by the NM_006259.3:c.1630G>T variant, which results in an in-frame deletion of these amino acids. **(E)** Amino acid sequence alignment of the altered PRKG2 region.

WGS in the proband revealed two previously unreported heterozygous variants in the *PRKG2* gene: a frameshift variant, NM_006259.3:c.1074del (p.Ala359LeufsTer24) (ClinVar submission number: VCV004537385.1), and a missense variant, NM_006259.3:c.1630G>T (p.Asp544Tyr) (ClinVar accession number: VCV004082151.1). Both variants are absent from the population database gnomAD v4.1.0. Since homozygous and compound-heterozygous variants in *PRKG2* have been described in patients with clinical features of acromesomelic dysplasia type 4 (Pagnamenta et al., 2022; Akgun-Dogan et al., 2024), both variants were reported. The NM_006259.3:c.1074del (p.Ala359LeufsTer24) variant was classified as likely pathogenic (LP) based on ACMG 2015 criteria (PM2 and PVS1). The novel NM_006259.3:c.1630G>T variant leads to the p.(Asp544Tyr) missense substitution, which has an AlphaMissense pathogenicity score of 0.671. In accordance with the ACMG/AMP 2015 guidelines, this variant is classified as being of uncertain clinical significance (VUS). Subsequent segregation analysis revealed the transposition of the variants (Figure 2A).

To clarify the pathogenicity of the NM_006259.3:c.1630G>T (p.Asp544Tyr) variant, we performed RNA analysis to assess its impact on the structure and expression of *PRKG2* mRNA. Preliminary bioinformatic analysis using SpliceAI predicted that this variant is likely to create a novel donor splice site (Δ score: 0.59), leading to a 6-nucleotide truncation of exon 13. We developed an RT-PCR system designed to amplify the *PRKG2* mRNA locus spanning exons 7–14, thus including both variants identified in the proband. We isolated RNA from fibroblast cultures of the proband and the parents and performed RT-PCR followed by targeted deep sequencing.

Analysis of the proband’s father revealed aberrant transcripts, which were absent in controls and characterized by a 6-nucleotide truncation of exon 13 (Figure 2B). Together with *in silico* predictions, these data demonstrate that the NM_006259.3:c.1630G>T variant indeed activates a cryptic splice donor site. This aberrant splicing results in an in-frame deletion of two amino acids, p.(Asp544_Arg545del). Following аnalysis of read coverage at the variant locus demonstrated that exon truncation occurred in approximately half of the transcripts carrying the NM_006259.3:c.1630G>T allele, whereas the remaining transcripts were expressed with the missense substitution p.(Asp544Tyr) (Figure 2C).

To assess the impact of the two-amino acid deletion (p.Asp544_Arg545del) and the missense substitution (p.Asp544Tyr) on *PRKG2* function, we performed a phylogenetic conservation analysis of the ASP544 residue by using ‘COBALT’ server (Papadopoulos and Agarwala, 2007) (Figure 2D). The results confirm that this residue is highly conserved across vertebrates, underscoring its functional importance. Moreover, we performed a 3D structural analysis using AlphaFold2 (Figure 2E). Both events affect an alpha-helix within the protein kinase domain and are predicted to disrupt its function. Based on our findings, we classified this variant as Likely Pathogenic and submitted it to ClinVar.

Investigation of the NM_006259.3:c.1074del (p.Ala359LeufsTer24) variant in the proband’s mother’s mRNA revealed a significant allelic imbalance (wt/mut ratio = 92/8) (Figure 2D). The marked reduction in the expression of the mutant allele is evidently due to the degradation of the majority of its transcripts via the nonsense-mediated decay (NMD) pathway. Thus, the vast majority of transcripts carrying the NM_006259.3:c.1074del (p.Ala359LeufsTer24) variant (∼45% of total transcripts) are subject to degradation. The small fraction (∼5% of total transcripts) that escapes NMD is expected to be translated into a protein truncated by 381 amino acids, p.(Ala359LeufsTer24).

## 4 Discussion

The present case expands the known phenotypic spectrum of *PRKG2*-related skeletal dysplasias, a group for which only a limited number of variants have been described in patients with an acromesomelic dysplasia phenotype. From both clinical and radiological perspectives, our findings align with and broaden the understanding of these disorders, which share signs of an acromesomelic pattern (Pagnamenta et al., 2022). As postulated by Mollaoğlu et al., the term “acromesomelic” is warranted for *PRKG2*-related dysplasia (Pagnamenta et al., 2022; Mollaoğlu et al., 2023).

Although the dysplastic features in our patient were generally mild, she exhibited disproportionate short stature with notably short forearms and hands. Radiographs revealed characteristic slender femoral necks with a biconcave and elongated appearance. A similar slender and biconcave morphology was observed in the second to fourth metacarpals. An elongated styloid process was another notable finding, a feature also visible in radiographs from previously published cases (Díaz-González et al., 2022). While pseudoepiphyses of the metacarpal bones were not described in earlier publications on this specific disorder, they are not unique to NPR-B-related skeletal dysplasias (Kenis et al., 2021) and may be linked to shared mechanisms of disturbed growth and ossification.

According to Kenis et al. (2021) and Díaz-González et al. (2022), the clinical and radiological similarities between these conditions are logical (Table 1). This is mechanistically supported, as Acromesomelic dysplasia, Maroteaux type is caused by biallelic variants in the gene encoding NPR-B, the main receptor for C-type Natriuretic Peptide (CNP), which acts upstream of cGKII (encoded by *PRKG2*). Our findings are consistent with previously published data and underscore the importance of a deeper understanding of the underlying mechanisms of skeletal growth and development in clinical practice.

**TABLE 1:** Brief clinical description of acromesomelic dysplasia types.

Type	Gene	Key clinical features	Radiographic findings
Acromesomelic dysplasia, type Maroteaux	*NPR2*	Adult short stature (<120 cm), shortened forearms, lower legs, hands, and feet, and normal intelligence (Faivre et al., 2000)	Broad fingers, square flat feet, and shortening of the long bones. The radius is bowed; the ulna is shorter than the radius. The skull is dolichocephalic (Faivre et al., 2000)
Acromesomelic dysplasia, type Hunter-Thompson	*GDF5*	Adult size: 100–130 cm. Skeletal abnormalities restricted to the limbs; the craniofacial skeleton and axial skeletal structures are normal. The hands and feet are most severely affected, but the distal phalanges are relative normal (Thomas et al., 1996)	Mesomelia and acromelia with shortening and deformity of the long bones, butterfly vertebrae, and narrowing of the interpedicular distances
Acromesomelic dysplasia, type Grebe	*GDF5* *BMPR1B*	Adult size: 100 cm. An extremely severe form with pronounced deformities and aplasia of the bones in the hands and feet. The fingers and toes lack articulation and appear as skin appendages. Axial skeletal structures and the craniofacial skeleton are not affected (Thomas et al., 1997)	Severely shortened limbs, multiple segmentation anomalies, aplasia/hypoplasia of the forearm and lower leg bones, and severe deformities of the hands and feet
Acromesomelic dysplasia, type Du Pan	*GDF5* *BMPR1B*	Normal head and trunk, hypoplastic or dysplastic or absent fibulae, and severe hypoplastic or dysplastic hand/feet abnormalities (Szczaluba et al., 2005)	Hypoplasia or aplasia of the tibia, shortening of the radius and ulna, and hand/foot anomalies, including polydactyly
Acromesomelic dysplasia-4 (summary of previously published cases)	*PRKG2*	Disproportionate short stature due to mesomelic shortening of the limbs (Díaz-González et al., 2022)	Radiographic hallmarks include mild to moderate platyspondyly, moderate brachydactyly, iliac flaring, and metaphyseal alterations of the long bones that progressively increase with age
Acromesomelic dysplasia-4 (current patient)	*PRKG2*	Borderline short stature with mild disproportion due to acro- and mesomelic shortening of the limbs Unger et al. (2023).	Mild brachydactyly with relatively longer great toes, no iliac flaring and metaphyseal alterations of the long bones, slender and biconcave appearance of the femoral necks and II-IV metacarpals, elongated styloid process of the ulna

Initial DNA diagnostics using a skeletal dysplasia gene panel (166 genes) failed to identify causative variants. This is explained by the fact that the first articles linking pathogenic *PRKG2* variants to acromesomelic dysplasia emerged only in 2021, while our patient’s panel analysis was conducted in 2022, prior to the gene’s inclusion.

The patient’s unique clinical presentation may be attributed to a distinct molecular disease mechanism. We identified a cryptic splice site variant in *PRKG2* with dual molecular consequences. This variant leads to the production of two aberrant transcript populations: one undergoes aberrant splicing, resulting in an in-frame deletion of two amino acids (p.Asp544_Arg545del), while the other is spliced normally but contains a missense substitution (p.Asp544Tyr).

The functional importance of this region, specifically of the Asp544 residue, is underscored by its high conservation across vertebrates, as revealed by phylogenetic analysis. Our 3D modeling demonstrated that both variants are located within an alpha-helical region of the kinase domain. To understand how these variants affect the kinase domain structure, a model of the mutant protein is required. However, the current AlphaFold model is trained and optimized for wild-type proteins; therefore, using it to simulate mutant proteins would not be reliable. We hypothesize that these alterations cause a partial, rather than complete, loss of protein function, i.e., they exert a hypomorphic effect on the protein’s structure and function. This mechanism potentially explains the patient’s attenuated clinical phenotype.

## 5 Conclusion

This paper describes the clinical and genetic features of a patient with mild acromesomelic dysplasia caused by novel compound-heterozygous variants in the *PRKG2* gene. Our findings expand the genetic and phenotypic spectrum of *PRKG2*-related disorders and underscore the critical role of functional studies in variant interpretation and establishing accurate genotype-phenotype correlations.

## Data Availability

The raw data supporting the conclusions of this article will be made available by the authors, without undue reservation.
